# Evaluation of the Influence of Process Parameters on the Properties of Resveratrol-Loaded NLC Using 2^2^ Full Factorial Design

**DOI:** 10.3390/antiox8080272

**Published:** 2019-08-03

**Authors:** Andréa A. M. Shimojo, Ana Rita V. Fernandes, Nuno R. E. Ferreira, Elena Sanchez-Lopez, Maria H. A. Santana, Eliana B. Souto

**Affiliations:** 1 Department of Engineering of Materials and Bioprocesses-School of Chemical Engineering, University of Campinas, Campinas 13083-970, Brazil; 2 Department of Pharmaceutical Technology, Faculty of Pharmacy, University of Coimbra, 3000-548 Coimbra, Portugal; 3 CQ Pharma, (FFUC), Pólo das Ciências da Saúde, Azinhaga de Santa Comba, 3000-548 Coimbra, Portugal; 4 Department of Pharmacy, Pharmaceutical Technology and Physical Chemistry, Faculty of Pharmacy, and Institute of Nanoscience and Nanotechnology (IN2UB), University of Barcelona, 08007 Barcelona, Spain; 5 Networking Research Centre of Neurodegenerative Disease (CIBERNED), Instituto de Salud Juan Carlos III, 28049 Madrid, Spain; 6 CEB-Centre of Biological Engineering, University of Minho, Campus de Gualtar, 4710-057 Braga, Portugal

**Keywords:** resveratrol, nanostructured lipid carriers (NLC), factorial design, high shear homogenization, ultrasound method

## Abstract

Resveratrol (RSV) is a natural antioxidant commonly found in grapes, berries, and nuts that has shown promising results in the treatment of a variety of degenerative and age-related diseases. Despite the proven beneficial results on reduction of reactive oxidant species (ROS) and on inflammatory process, RSV shows various limitations including low long-term stability, aqueous solubility, and bioavailability, restricting its applications in the medical-pharmaceutical area. To overcome these limitations, it has been applied in pharmaceutical formulations as nanostructured lipid carriers (NLC). Thus, the present study focuses on the optimization of the production process of NLC. NLC was produced by high shear homogenization (HSH) and ultrasound method (US) using Compritol^®^ ATO C888 as solid lipid and Miglyol 812^®^ as liquid lipid. In order to obtain an optimized formulation, we used a 2^2^ full factorial design with triplicate of central point investigating the effects of the production process parameters; shear intensity and homogenization time, on the mean particle size (PS) and polydispersity index (PDI). Instability index, encapsulation efficiency, and production yield were also evaluated. As the PS and PDI values obtained with 6 min of shear at 19,000 rpm and 10 min of shear and 24,000 rpm were similar, the instability index (<0.1) was also used to select the optimal parameters. Based on the results of the experimental design and instability index, it was concluded that the shear rate of 19,000 rpm and the shear time of 6 min are the optimal parameters for RSV-loaded NLC production. Factorial design contributed therefore to optimize the variables of the NLC production process from a small number of experiments.

## 1. Introduction

Reactive oxygen species (ROS) are directly associated with a variety of degenerative and age-related diseases, and other pathologies, including different types of cancers. ROS are generated as by-products of cellular metabolism and their excessive production can damage lipids, proteins, and DNA, different cells, and tissues [1].

Resveratrol (RSV) (trans-3,4,5-trihydroxystilbene) is a natural antioxidant commonly found in grapes, berries, and nuts that has shown promising results in the treatment of a variety of degenerative and age-related diseases. It blocks the activation of nuclear factor κB (NF-κB), reducing the generation of ROS and pro-inflammatory cytokines (interleukin IL-1 β and IL-6), which results in the inhibition of chondrocyte apoptosis, inflammation, and the progression of several diseases [2,3]. It is also a direct inhibitor of cyclooxygenase 2 (COX-2) which produces pro-inflammatory lipid mediators (leukotrienes and prostaglandins) responsible for pain sensation [4].

In recent years, RSV has also shown beneficial results in modulation of tissue regeneration, microcirculation, the function of peripheral nerves, production of anti-inflammatory cytokines, and insulin [5,6,7,8,9]. However, RSV shows low long-term stability, rapid metabolism, and release; low aqueous solubility (0.05 mg/mL) and bioavailability. RSV is also unstable under the influence of light, certain pH levels, and temperature, which causes isomerization or degradation of RSV, making it difficult to apply in the medical–pharmaceutical area [10,11,12,13]. To overcome these limitations, RSV has been employed in pharmaceutical formulations in different drug delivery systems (DDS), including microparticulate system [14,15], micro/nanocapsules [16], cyclodextrin complexes [17,18], solid lipid nanoparticles (SLN) [19,20,21,22], nanosuspensions [23], vesicular systems liposomes [24], niosomes [25], nanosponges [18,26], microspheres [27], transfersomes and ethosomes [14,15,28], and nanostructured lipid carriers (NLC) [19,20]. The DDS are employed to improve the physicochemical stability of loaded drugs, provide a sustained-release profile, increase plasma half-life, decrease the risk of immunogenicity, improve the drug solubility and thereby its bioavailability and therapeutic activity, enhance antioxidant activity, and improve the permeation and targeted delivery [14,15].

NLC consists of a mixture of solid and liquid lipids, which creates an imperfect crystalline structure, providing more space between the lipid chains and the matrix [29,30]. The main advantages of the use of NLC are high encapsulation efficiency and storage stability, the possibility of controlling the release of several drugs, low toxicity due to the absence of solvents in the production process, and the possibility of production in an industrial scale [31,32]. Moreover, Gokce et al. [14] observed that RSV-loaded NLC penetrated deeper into the skin [19]. Jose et al. showed a negligible release of resveratrol over several hours, corroborating the high stability of RSV-loaded NLC [20].

Experimental designs have been the most used tools to simultaneously analyze the influence of different variables on the properties of NLC, aiming to ensure the high product quality, the economy of production, and reduction of production time, allowing the scale-up of the process [33].

To evaluate the optimum experimental conditions for NLC production, several authors have assessed the influence of different factors which can affect the final properties of formulations, including type, ratio, and concentration of lipids and stabilizers, cycle numbers, time and intensity of homogenization, and pressure [34,35,36]. Thus, this work reports the effects of the production process parameters, shear intensity, and homogenization time of RSV-loaded NLC by means of a 2^2^ factorial design with triplicate of the central point, measuring the mean particle size (PS) and polydispersity index (PDI) as the dependent variables.

## 2. Materials and Methods

### 2.1. Materials

Hyaluronic acid sodium salt (HA, MW = 1.01–1.8 MDa) was purchased from Lifecore Biomedical Co. (Chaska, MN, USA). Resveratrol (RSV) was obtained from Sigma-Aldrich Co. (St. Louis, MO, USA). Tween^®^ 80 (Polysorbate 80) was purchased from Uniqema (Everberg, Belgium). Compritol^®^ ATO C-888 was purchased from Gattefossé (Nanterre, France) and Miglyol 812^®^ from Sasol Chemical Industries Ltd. (Homburg, Germany). Poloxamer^®^ 188 (Pluronic F68, Kolliphor^®^ P188) was obtained from BASF (Ludwigshafen, Germany). All other reagents and solvents were analytical grade and used exactly as received. Ultra-purified water was obtained from Milli-Q^®^ Plus system, home supplied.

### 2.2. Preparation of RSV-Loaded NLC

RSV-loaded NLC were prepared through the high shear homogenization method associated with sonication [19,20]. The lipid phase was composed of 255 mg Compritol^®^ 888 ATO (C-888), 45 mg Miglyol 812^®^, and 10 mg RSV, and the aqueous phase was composed of 150 mg of Poloxamer^®^, 188 (P188), and 75 mg of Tween^®^ 80 (Tw 80) in 12.5 mL bidistilled water were heated at 85 °C, separately. The aqueous phase was poured into the lipid phase and stirred with Ultra Turrax homogenizer Ystral GmbH X10/25 (Dottingen, Germany), followed by sonication for 15 min at 70% intensity (14 watts) using a Sonics and Materials Vibra-Cell™ CV18 (Newtown, CT, USA), according to Table 1. The particles were dispersed in 12.5 mL of bidistilled water and kept at −20 °C for 10 min. Blank (i.e. non-loaded NLC) was prepared in a similar way, without the RSV. The formulations were stored at 4 °C.

### 2.3. 2^2^ Factorial Design 

The influence of the shear intensity and homogenization time on the NLC properties was evaluated using a 2^2^ factorial design with triplicate runs of the central point to estimate the experimental error, composed of 2 variables, which were set at 2-levels each (Table 2). The mean particle size and polydispersity index (PDI) were the dependent variables. The design required a total of 7 experiments. Each factor, the lower and higher values of the lower and upper levels, was represented by a (−1) and a (+1), and the central point was represented by (0), as summarized in Table 1. The data were analyzed using STATISTICA 7.0.

### 2.4. Characterization of RSV-Loaded NLC

#### 2.4.1. Particle Size and Polydispersity Index

The particle size (PS) and polydispersity index (PDI) of NLC were measured at 25 °C using photon correlation spectroscopy (PCS) (dynamic light scattering, DLS, Zetasizer Nano NS, Malvern Instruments, Malvern, Worcs, UK). The measurements were carried out using a He–Ne laser at 633 nm and 4.0 mW power, with a back-scattering detection angle of 173° after dilution of formulations with ultra-purified water. The average hydrodynamic diameter was recorded based on the observed diameters weighted by the number size distribution. The polydispersity index (PDI) was also calculated from cumulative analysis of the measured DLS intensity autocorrelation function (a dimensionless number that ranges from 0 to 1). PS and PDI of NLC were determined in triplicate. For each measurement, the NLC was diluted in Milli-Q^®^ water to an appropriate concentration to avoid multiple scattering.

#### 2.4.2. Zeta Potential

The zeta potential was determined by applying an electric field across the samples, and the value of the zeta potential was obtained by measuring the velocity of the electrophoretic mobility of the particles using the laser Doppler anemometry technique. The measurements were performed in triplicate for each sample at 25 °C using a Malvern Zetasizer Nano ZS (Malvern Instruments, Worcs, UK). Milli-Q^®^ water was used to dilute the NLC to a proper concentration. The zeta potential was calculated using the Helmholtz-Smoluchowsky equation included in the software of the system. Values are presented as the mean of triplicate runs per sample.

#### 2.4.3. Stability Index

Analytic centrifuge LUMiSizer (LUM GmbH, Dias de Sousa, Portugal), which accelerates the destabilization phenomena, was used to evaluate the simulated long-term physical stability of NLC. Briefly, the samples without prior dilution were placed in rectangular test tubes (optical path of 2 mm) and exposed to centrifugal force at 10,000 rpm, measuring 300 profiles in intervals of 10 s at 25 °C. These experiments allowed differentiation between various instability mechanisms at an accelerated rate. Extrapolated results were used to estimate dispersion shelf life in minutes. To simply assess the physical stability of NLC, the instability index was calculated by the delivered software (SepView 6.0; LUM, Berlin, Germany). The index was quantified by the clarification at a given separation time, divided by the maximum clarification, according to Hoffmann and Schrader [37]. 

#### 2.4.4. Entrapment Efficiency

The drug entrapment efficiency (EE) was determined by UV-visible spectrometry at 303 nm, using the Synergy™ HTX Multi-Mode Microplate Reader (Biotek Instruments, Winooski, VT, USA). Briefly, NLC was placed in the dialysis bag (cutoff 14 kDa) (Sigma-Aldrich, St. Louis, MO, USA). Then, the bags were placed in centrifuge tubes, covered with a mixture of ethanol and water 1:1 (*v*/*v*), and centrifuged for 1.5 h at 5000 rpm in centrifuge Laborzentrifugen 3K15 (Sigma, Osterode am Herz, Germany). The mixture of ethanol and water was analyzed for RSV content through the standard curve allowing the quantity of free drug to be determined. The encapsulated amount of RSV was calculated by subtracting the free amount of RSV from the total amount present in the dispersion. The measurements were performed in triplicate. The EE percentage was calculated by the Equation (1).

(1) $$EE=\frac{\left(amount\;of\;initial\;RSV-amount\;of\;free\;RSV\right)}{amount\;of\;initial\;RSV}\;\times \;100$$

#### 2.4.5. Production Yield

The NLC formulations were frozen for 12 h at −80 °C and lyophilized at a pressure of 0.1 mbar for 24 h at −40 °C using a Telstar LyoQuest Freeze Dryer (Barcelona, Spain). The production yield (Y_NLC_) was calculated by Equation (2).

(2) $$Y_{NLC}\;\left(\%\right)=\frac{\left(initial\;amount-final\;amount\right)}{initial\;amount\;}\;\times \;100$$

#### 2.4.6. Morphology

The morphology was performed with a transmission electron microscope Tecnai G2 Spirit Biotwin (FEI Company, Eindhoven, The Netherlands). The samples were stained with 2% (*w*/*v*) phosphotungstic acid and placed on copper grids for viewing by transmission electron microscopy (TEM).

### 2.5. Statistical Analysis

Statistical differences were determined using analysis of variance (ANOVA), followed by Tukey’s test for comparisons between groups. The significance level was taken as 95% (*p* < 0.05). Factorial design data were analyzed using STATISTICA 7.0.

## 3. Results and Discussion

The lipid composition and concentration used in the preparation of NLC were chosen based on work reported by Gokce et al. [19] in which the optimal liquid lipid concentration (Miglyol 812^®^) was 15% of the whole lipid phase (Compritol^®^ 888 ATO and Miglyol 812^®^) [19]. Poloxamer^®^ 188 and Tween^®^ 80 were used as stabilizers of the formulations. Compritol^®^ 888 ATO is a solid lipid composed of glycerol tribehenate (28–32%), glycerol dibehenate (52–54%), and glycerol monobehenate (12–18%). The main fatty acid is behenic acid (C_22_) (>85%), but other fatty acids (C_16_–C_20_) are also present [38]. Miglyol 812^®^ is a medium chain triglyceride composed mainly of caprylic (C8:0; 50–80%) and capric (C10:0; 20–50%) fatty acids with a minor level of caproic (C6:0; ≤2%), lauric (C12:0; ≤3%), and myristic (C14:0; ≤1%) fatty acids [39]. 

Mohammadi et al. [39] and Kovasevic et al. [40] demonstrated that Miglyol 812^®^ can be used in the range of 10–60% of total lipid without affecting the mean particle size and the distribution of NLC. 

The choice of stabilizers is also a very important step in the preparation of NLC formulations because they control the particle size and the stability, preventing their aggregation during storage [39,41]. Currently, the non-ionic surfactants Poloxamer 188^®^ and Tween^®^ 80 are the most used for the preparation of these formulations [42]. According to Tamjidi et al. [42], the steric repulsion is the major colloidal interaction among NLC stabilized with non-ionic surfactants, yielding good stability to the variations of concentration and pH of electrolytes, and to the freeze–thaw stages. Moreover, non-ionic surfactants have lower toxicity and irritation potential than ionic ones [43]. 

However, NLC prepared with non-ionic surfactants may undergo a weak flocculation, as well as requiring large amounts of surfactants to cover the particle surface compared to those stabilized by electrostatic repulsion [44].

In Poloxamer^®^ 188, the hydrophobic polypropylene oxide chains are adsorbed onto the particle surface as the “anchor chain”, while the hydrophilic polyethylene oxide chains are pulled out from the surface to the aqueous medium, thereby creating a stabilizer layer [45]. In addition, Poloxamer^®^ 188 exhibits low toxicity, can control release and targeted delivery applications, and is stable at high temperatures [46]. Tween^®^ 80 is a polyethoxylated sorbitan and oleic acid derivative that has high surface activity and low toxicity [39]. The coating with Tween^®^ 80 improves the stability of the lipid present in NLC by hydration in the surface layer [47,48].

In this work, we used a combination of these surfactants because they produce a layer at the interface, generating high coverage as well as adequate viscosity to improve the stability and synergism in the particle size reduction [39]. Aiming to obtain particles in the nanometer range, NLC was produced by an association of high shear homogenization (HSH) [19] and ultrasound method (US) [20]. HSH produced particles in the micrometer range (pre-emulsion) and the ultrasound method reduced the microparticles to the nanometer range. 

The effects of the formulation variables (independent variables)-shear intensity and homogenization time on the response parameters (dependent variables)-mean particle size (PS) and polydispersity index (PDI), were evaluated using full factorial design 2^2^ with triplicate of the central point. For the factorial design study, a total of seven experiments were required. Zeta potential, encapsulation efficiency (EE), production yield (Y), and instability index were also measured.

Table 3 shows the influence of shear intensity and homogenization time on NLC production (RSV-loaded NLC and NLC without RSV = placebo).

The combination of HSH and US methods produced placebos (NLC) with sizes ranging between 100 nm and 260 nm, and RSV-loaded NLC (NLC-RSV) with sizes ranging between 125 nm and 190 nm.

Particle size of less than 200 nm was attributed to the efficiency of the emulsion step. Gokce et al. [49] observed that the Compritol^®^ 888 ATO tends to return to solid form during mixing because this lipid is a mixture of mono-, di-, and triglycerides. It is known that the longer the fatty acid triglyceride, the higher the temperature needed to convert it from the solid state to liquid (melt) state. However, the presence of Miglyol^®^ 812 helps to distribute the heat energy more homogeneously due to the high concentration of unsaturated fatty acids reducing the melting point of the system. This results in a more efficient emulsification, which in turn has an effect on the size of the particles formed. After cooling, the pre-emulsion shows smaller particles, which may result in even smaller nanoparticles [50,51]. Thus, the stability is related to the lipid composition, since NLC presents a disordered lipid matrix conferred by the presence of liquid lipid and to polysorbate surfactant (Tween^®^ 80) used in its preparation [52]. All NLC formulations showed a PDI of above 0.2 and negative ZP around −12 mV.

The PDI has an important effect on the physical stability and uniformity (distribution) of NLC. The values should be as low as possible to ensure the long-term stability. PDI values of 0.1–0.25 show a narrow size distribution, while PDI values greater than 0.5 indicate a very broad distribution [53]. The PDI values obtained from placebo and RSV-loaded NLC above 0.2 indicated a non-monodisperse distribution with the presence of aggregated suggesting lower long-term stability. This type of distribution is usual in NLC produced using the HSH and US method, where it is very difficult to achieve a unimodal distribution of sizes [20].

ZP is also an indirect measurement of the long-term physical stability of NLC. It relates to the trend of particles to aggregate. According to Lakshimi and Kumar (2010), in electrostatically stabilized NLC, a good stability is achieved in ZP above ±30 mV, whereas in a combination of electrostatic and steric stabilization, a minimum of ZP of ±20 mV is desirable [53,54]. In addition, ZP of ±0–5 mV produces a maximum flocculation [32,55,56]. As shown in Table 3, all NLC had a negative ZP around −12 mV, indicating moderate stability regardless of RSV incorporation, suggesting that RSV did not significantly alter the ZP of the formulations (*p* > 0.05).

Besides the ZP, the long-term stability was also assessed by the instability index. The instability index is a dimensionless number between 0 (more stable) and 1 (more unstable), calculated based on the clarification at a given separation time, divided by the maximum clarification. For that, we used the LUMiSizer^®^ equipment, which allows the measurement of the transmitted light intensity during centrifugation, as a function of time and position, over the entire sample length [57,58].

In spite of the ZP values, with the exception of the NLC-RSV_2_ and NLC-RSV_4_ formulations, the dispersion analysis indicated a good simulated physical stability of the NLC containing RSV, expressed as instability index (<0.05). This observation suggests that these particles will remain stable and have a good dispersion quality in long-term storage.

The results of encapsulation efficiency showed that a large amount of RSV (EE > 92%) was incorporated in all RSV-loaded NLC formulations, suggesting its preferential partition into lipid matrix of the nanoparticles [15]. Gokce et al. [14] also obtained the EE of 91% using the same formulation. In addition, the production yield of both placebo and RSV-loaded NLC was found to be satisfactory, with an average above 60%.

**Figure 1:**
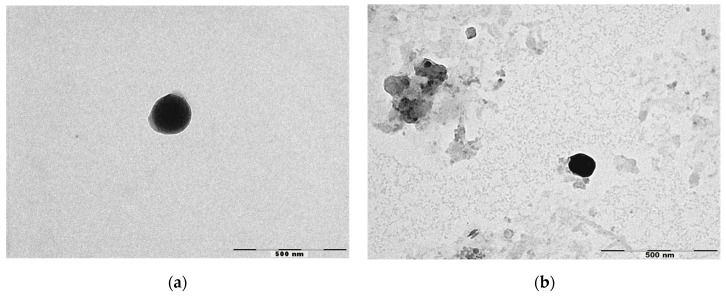
Micrographs obtained by TEM of (**a**) NLC_4_ and (**b**) NLC-RSV_4_. Scale bar = 500 nm.

Figure 1 shows the micrographs obtained by TEM of NLC and NLC-RSV. TEM analysis confirmed the colloidal sizes of particles. NLC was almost spherical and uniform in shape with smooth surfaces, while NLC-RSV showed more amorphous shapes. No crystallization of RSV was observed on the surface of NLC-RSV. Thus, our study suggests that the lipid matrix used solidified upon cooling, but it remained in the amorphous state, helping with the accommodation of RSV in a lipid matrix [38].

**Figure 2:**
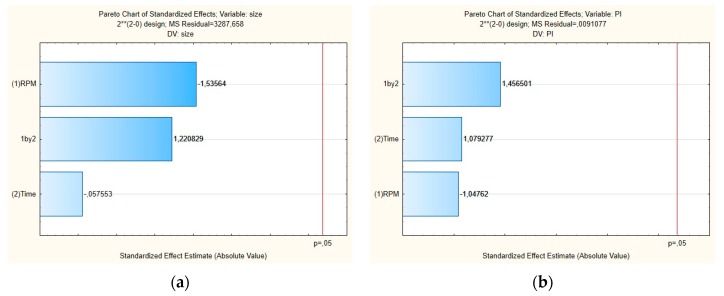
Pareto charts of the standardized effects for the placebo obtained for (**a**) particle size (Z-average) and (**b**) polydispersity index (PDI).

**Figure 3:**
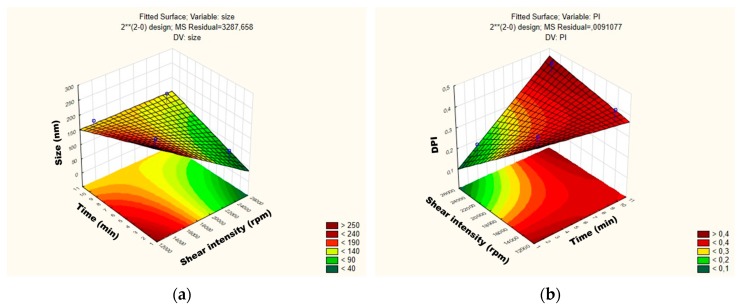
Surface response charts of experimental design of the placebo obtained for (**a**) particle size (Z-average) and (**b**) polydispersity index (PDI).

Figure 2 shows the Pareto chart of the standardized effects and Figure 3 shows the surface response charts of experimental design for the production of placebos. As shown in Figure 2a,b, the PS and their PDI were not significantly influenced by tested parameters; neither was the interaction between variables.

For the mean particle size, the p-value obtained by shear intensity was −1.53564, homogenization time was −0.57553, and the interaction was 1.220829, while for the PDI, the *p*-value obtained by shear intensity was −1.04762, homogenization time was 1.079277, and the interaction was 1.456501. These parameters and their interaction were reported not to be statistically significant. However, the response surface charts of experimental design (Figure 3a,b), show that increasing the shear intensity decreases the average size and the PDI. Moreover, in Figure 3, we observed that the average PS is slightly affected by the homogenization time, while PDI is not affected. 

Comparing NLC_1_ with NLC_2_ and NLC_3_ with NLC_4_, we observed two trends where the PS goes down in NLC_1_/NLC_2_ and where PS goes up in NLC_3_/NLC_4_ by increasing the homogenization time.

Thus, although neither variable is statistically significant when the placebos are subjected to a lower homogenization time and shear intensity, they tend to be larger, i.e., approximately 263 nm, and the PDI is >0.40. The placebo produced with shear intensity of 19,000 rpm and homogenization time of 6 min showed a smaller PS, around 105 nm. 

**Figure 4:**
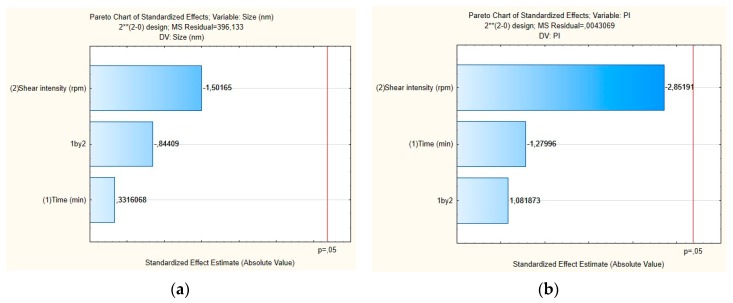
Pareto charts of the standardized effects for RSV-loaded NLC obtained for (**a**) particle size (Z-average) and (**b**) polydispersity index (PDI).

**Figure 5:**
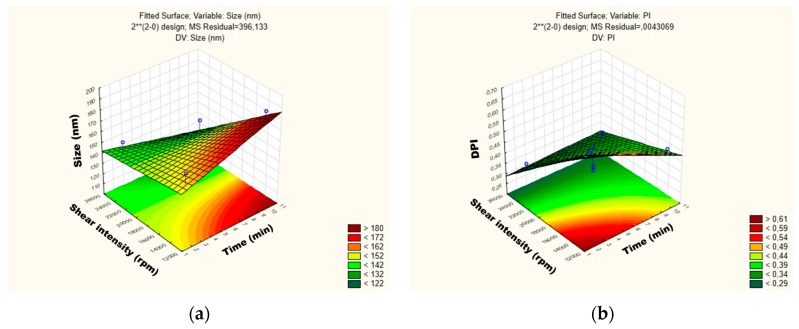
Surface response charts of experimental design of RSV-loaded NLC obtained for (**a**) particle size (Z-average) and (**b**) polydispersity index (PDI).

The influence of each independent variable and their interactions on RSV-loaded NLC were also evaluated by Pareto charts (Figure 4) and surface response (Figure 5). As shown in Figure 4a,b, the PS and their PDI were not significantly influenced by tested parameters; neither was the interaction between variables. For the mean particle size, the p-value obtained by shear intensity was −1.50165, homogenization time was 0.3316068, and the interaction was −0.84409, while for the PDI, the *p*-value obtained by shear intensity was −2.85191, homogenization time was −127996, and the interaction was 1.081873. These parameters and their interaction were reported not to be statistically significant. However, the response surface charts of experimental design (Figure 5a,b), shows that increasing the shear intensity decreases the average size and the PDI. Moreover, in Figure 5, we observed that both particle size and PDI are slightly affected by the homogenization time. Thus, although neither variable is statistically significant when the RSV-loaded NLC are subjected to a smaller homogenization time and intensity shear, the PDI is >0.54. We observed that smaller particles are obtained by increasing shear intensity. However, comparing NLC-RSV_1_ with NLC-RSV_2_ and NLC-RSV_3_ with NLC-RSV_4_, we observed two trends where the PS goes up in NLC-RSV_1_/NLC-RSV_2_ and where PS goes down in NLC-RSV_3_/NLC-RSV_4_ by increasing the homogenization time.

The RSV-loaded NLC produced at the central point with the shear intensity of 19,000 rpm and homogenization time of 6 min showed a smaller PS, around 135 nm.

As the experimental results of PS and PDI of NLC-RSV_4_ were similar to the results obtained for NLC-RSV_5_, NLC-RSV_6_, and NLC-RSV_7_, we also used the instability index to select as optimal parameter.

Thus, based on the results of the experimental design and instability index, it was concluded that the shear rate of 19,000 rpm and the shear time of 6 min are the optimal parameters for RSV-loaded NLC production.

## 4. Conclusions

This study attempted to design and optimize RSV-loaded NLC prepared by a combination of high shear homogenization and ultrasound method. After selecting the critical process variables affecting particle size (PS) and polydispersity index (PDI), a 2^2^ factorial design with triplicate of the central point was employed to plan and perform the experiments. Zeta potential, morphology, drug entrapment efficiency, production yield, and stability index were also measured. RSV-loaded NLC and NLC without RSV (placebo) were prepared. Optimized NLC formulation was prepared based on the predicted optimum levels of the independent variables, shear intensity, and homogenization time of the factorial design using Pareto charts and surface response charts, and on instability index. Thus, optimal parameters for NLC were obtained using shear intensity of 19,000 rpm and shear time of 6 min, producing NLC with PS around 135 nm and DPI around 0.4. Moreover, these production process parameters produced particles with high entrapment efficiency (~93%) and production yield (~65%).

## Figures and Tables

**Table 1:** Preparation of resveratrol-loaded nanostructured lipid carriers (NLC). Factorial design, providing the lower (−1), upper (+1), and (0) central point level values for each variable.

Factor	Coded Levels		
	−1	0	+1
Shear intensity (rpm)	13,000	19,000	24,000
Shear time (minutos)	2	6	10

**Table 2:** A 2^2^ full factorial experimental design layout. The formulation codes of NLC with resveratrol are named as NLC-RSV_(number of the experiment)_ and the one without resveratrol as NLC_(number of the experiment)_.

Formulation Code	Coded Factor Level	
	Factor 1	Factor 2
NLC_1_ or NLC-RSV_1_	−1	−1
NLC_2_ or NLC-RSV_2_	+1	−1
NLC_3_ or NLC-RSV_3_	−1	+1
NLC_4_ or NLC-RSV_4_	+1	+1
NLC_5_ or NLC-RSV_5_	0	0
NLC_6_ or NLC-RSV_6_	0	0
NLC_7_ or NLC-RSV_7_	0	0

**Table 3:** Influence of shear intensity and homogenization time on the production of NLC.

	Shear Time (minutes)	Shear Intensity (rpm)	PS ± SD (nm)	PDI ± SD	Zeta ± SD (mV)	Entrapment Efficiency (%)	Production Yield (%)	Instability Index *
NLC_1_	2	13,000	263.6 ± 3.9	0.465 ± 0.020	−12.97 ± 0.50	-	97 ± 2	0.022
NLC_2_	10	13,000	190.3 ± 1.8	0.429 ± 0.025	−10.02 ± 0.24	-	78 ± 4	0.044
NLC_3_	2	24,000	111.6 ± 1.1	0.236 ± 0.001	−13.93 ± 0.47	-	73.5 ± 0.6	0.040
NLC_4_	10	24,000	178.3 ± 1.6	0.478 ± 0.022	−12.03 ± 0.51	-	66 ± 1	0.034
NLC_5_	6	19,000	109.1 ± 0.7	0.265 ± 0.008	−10.03 ± 0.23	-	71 ± 3	0.043
NLC_6_	6	19,000	103.4 ± 1.5	0.279 ± 0.036	−11.00 ± 0.46	-	68 ± 13	0.03
NLC_7_	6	19,000	106.4 ± 0.1	0.271 ± 0.006	−12.83 ± 0.35	-	65.8 ± 0.9	0.032
NLC-RSV_1_	2	13,000	164.3 ± 3.2	0.619 ± 0.010	−13.23 ± 0.21	94.6 ± 0.4	76.1 ± 0.9	0.073
NLC-RSV_2_	10	13,000	187.7 ± 0.9	0.464 ± 0.003	−8.43 ± 0.15	94.4 ± 0.1	78 ± 2	0.493
NLC-RSV_3_	2	24,000	153.1 ± 1.0	0.366 ± 0.005	−12.07 ± 0.40	96.3 ± 0.1	72 ± 3	0.041
NLC-RSV_4_	10	24,000	142.9 ± 2.5	0.353 ± 0.016	−11.67 ± 0.70	94.2 ± 0.1	69 ± 1	0.244
NLC-RSV_5_	6	19,000	125.3 ± 0.5	0.357 ± 0.009	−12.87 ± 0.38	92.9 ± 0.4	67.9 ± 0.6	0.024
NLC-RSV_6_	6	19,000	139.4 ± 0.7	0.339 ± 0.006	−12.90 ± 0.27	93.15 ± 0.04	62 ± 4	0.025
NLC-RSV_7_	6	19,000	146.6 ± 3.2	0.444 ± 0.061	−13.00 ± 0.53	92.0 ± 0.3	63 ± 11	0.011

SD = standard deviation; PDI = polydispersity index. * Instability index measured at t = 200 s.
